# Analysis of the primary factors influencing donor derived cell-free DNA testing in kidney transplantation

**DOI:** 10.3389/fimmu.2024.1435578

**Published:** 2024-09-06

**Authors:** Changling Cao, Li Yuan, Yinfeng Wang, Haitao Liu, Haider Cuello Garcia, Huiqiang Huang, Weiqiang Tan, Yang Zhou, Haifeng Shi, Tingya Jiang

**Affiliations:** ^1^ Biostatistics, Research & Development (R&D), AlloDx Biotech (Shanghai), Co., Ltd, Shanghai, China; ^2^ Xiang’an Hospital of Xiamen University, School of Medicine, Xiamen University, Xiamen, China; ^3^ School of Life Sciences, Jiangsu University, Zhenjiang, China; ^4^ Medical Department, AlloDx Biotech (Shanghai), Co., Ltd, Shanghai, China

**Keywords:** donor derived cfDNA, SNP counts, sequencing depth, absolute and fraction value, nucleosome foot printing

## Abstract

The donor-derived cell-free DNA (ddcfDNA) is found in the plasma and urine of kidney transplant recipients and displays notable potential in diagnosing rejection, specifically antibody-mediated rejection (ABMR). Nonetheless, the quantitative methods of ddcfDNA lacking standardization and diverse detection techniques can impact the test outcomes. Besides, both the fraction and absolute values of ddcfDNA have been reported as valuable markers for rejection diagnosis, but they carry distinct meanings and are special in various pathological conditions. Additionally, ddcfDNA is highly sensitive to kidney transplant injury. The various sampling times and combination with other diseases can indeed impact ddcfDNA detection values. This review comprehensively analyses the various factors affecting ddcfDNA detection in kidney transplantation, including the number of SNPs and sequencing depths. Furthermore, different pathological conditions, distinct sampling time points, and the presence of complex heterologous signals can influence ddcfDNA testing results in kidney transplantation. The review also provides insights into ddcfDNA testing on different platforms along with key considerations.

## 1 Introduction

Current diagnostic measures are unreliable in the early detection of kidney graft injury, including acute, chronic and subclinical rejection. Changes in plasma creatinine are most commonly used to assess the function of transplanted kidney. However, plasma creatinine is a result of glomerular function rather than renal tissue damage with low specificity and hysteresis. By the time an increase in plasma creatinine related to rejection is evident, a significant degree of tissue damage has already occurred within the kidney. Biopsy is regarded as the “gold standard” for diagnosing graft injury. However, it is not a suitable method because of its prohibitive cost and potentially serious complications. In addition, sampling and interpretation errors can also affect the accuracy of a biopsy.

Previous studies have evaluated the clinical validity of ddcfDNA as a non-invasive biomarker for comprehensive monitoring of allograft injury, including in kidney transplantation (KTx) (1–3). It has shown great promise in the diagnosis of kidney transplant rejection, particularly ABMR. This new approach could be useful in personalizing immunosuppression and thereby improving outcomes.

The current methods of quantifying ddcfDNA concentration are primarily based on Next-generation sequencing (NGS) or PCR technologies, including droplet digital PCR (ddPCR) and real-time PCR. These methods calculate the fraction of ddcfDNA by assessing the proportion of low-frequency heterologous signals (LFHS) on the homozygote single nucleotide polymorphisms (SNPs) of recipients. Previous studies have used Y-chromosome specific alleles to quantify the ddcfDNA, but this technique could only be performed in gender-mismatched transplantation settings (4). The application of ddPCR is limited by the number of SNP sites, and detection results are prone to bias. It is important to note that various detection techniques can influence test results to some extent (1).

Furthermore, both the fraction and absolute value of ddcfDNA have been reported as valuable markers for the diagnosis of rejection. However, they carry distinct meanings and are specific to different pathological conditions (e.g., lung or urinary tract infection, parvovirus B19 infection, TCMR), which may complicate interpretation of the results. For example, in some situations the absolute ddcfDNA value may increase but the fraction value remains stable. Cheng et al. have shown that the ddcfDNA fraction is higher in ABMR than that in TCMR (5). Previous studies identified the association between ddcfDNA and renal allograft injury, which found that ddcfDNA level has an obvious different manifestation in various renal allograft injuries (6). Moreover, inflammatory load and macrophage extracellular trap activity can increase the absolute values of ddcfDNA, but decrease the plasma ddcfDNA fraction (7).

Lastly, ddcfDNA is very sensitive to renal transplant injury, different sampling times (early post-transplant, post-steroid pulse, within 12 h of biopsy, etc.) and the presence of third heterologous signals (such as tumor carriage or transfusion status) can indeed affect ddcfDNA detection values. The median ddcfDNA level increases at the initial time post-transplantation and remains at a stable level till the seventh day post-transplantation (8). Moreover, ddcfDNA test values are also elevated during the immediate post-puncture period (9). While there is a significant decrease in plasma ddcfDNA levels after rejection treatment (10), urine ddcfDNA levels show a distinct pattern in BK virus-associated nephropathy (BKVAN) patients (11). Therefore, it is crucial to adhere to precise sampling requirements and select appropriate sampling times to ensure accurate interpretation of ddcfDNA results.

This article provides a comprehensive review of various detection technology platforms, different pathological conditions, distinct sampling time points, and the presence of complex heterologous signals, and how can they influence ddcfDNA testing results in kidney transplantation. It also provides insights into ddcfDNA testing in different platforms with important considerations.

## 2 Different testing technology platforms

### 2.1 Donor genotype-dependent and -independent methods

**Figure 1:**
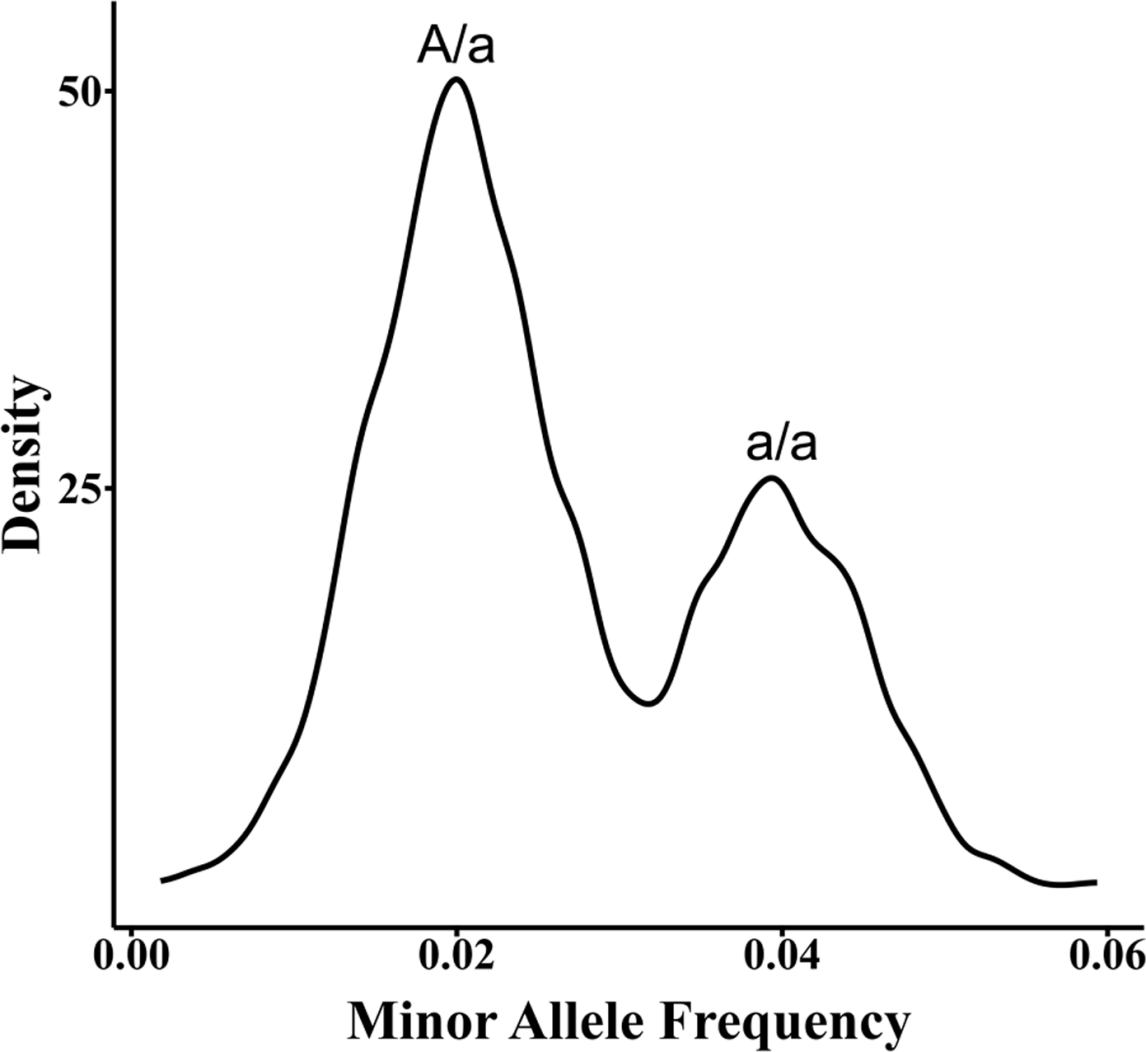
Two signal distribution of a/a and A/a.

Differentiating donor and recipient signals primarily relies on genetic markers like SNP and insertion-deletion (Indel). Donor genotype-dependent methods require prior acquisition of both recipient and donor genotype information to accurately identify and quantify donor signals within the recipient’s cfDNA sample (12). However, in cases where the donor genotype is unavailable, direct calculation of the donor proportion is not possible as it is challenging to determine whether LFHS at SNP positions are erroneous, derived from the donor, or represent donor homozygous or heterozygous signals. To address the limitation of unavailable donor genotype information, the first step is to determine the recipient’s genotype. This can be done by analyzing leukocyte samples obtained from the low-depth cfDNA WGS data (13, 14), or ddPCR data (15), or by inferring the recipient’s SNP genotype through deep sequencing (16–18).Once the recipient’s genotype is established, informative SNPs are identified. These are SNPs where the recipient is homozygous, and LFHS are observed. The probabilities of donor homozygous and heterozygous genotypes at these informative SNPs are calculated based on the SNP genotype frequencies in the population and the law of independent assortment. These probabilities are then incorporated into a binomial distribution probability model. Finally, the quantification of ddcfDNA is achieved by applying the maximum likelihood estimation method (18). For example, when the recipient genotype at the informative SNP locus is “A/A”, and a minor signal “a” is detected, there are two possibilities for the “a” signal: a/a or A/a. In addition, hundreds or thousands of such low signals will form two signal proportions (**Figure 1**) and conform to two laws: the signal values of a/a are twice as much as that of A/a and the number of SNPs in the form of a/a is half of that in the A/a form.

Additionally, “a” deviates from this law due to PCR or sequencing errors (18). Donor-independent quantification relies on the assumption that ddcfDNA content does not exceed 20% or 15% (19). To quantify ddcfDNA in the early post-transplantation period (<8 days), simultaneous determination of the recipient leukocyte genotype is critical, as the ddcfDNA often exceeds 20% (8).

### 2.2 The impact of nucleosome footprinting

**Figure 2:**
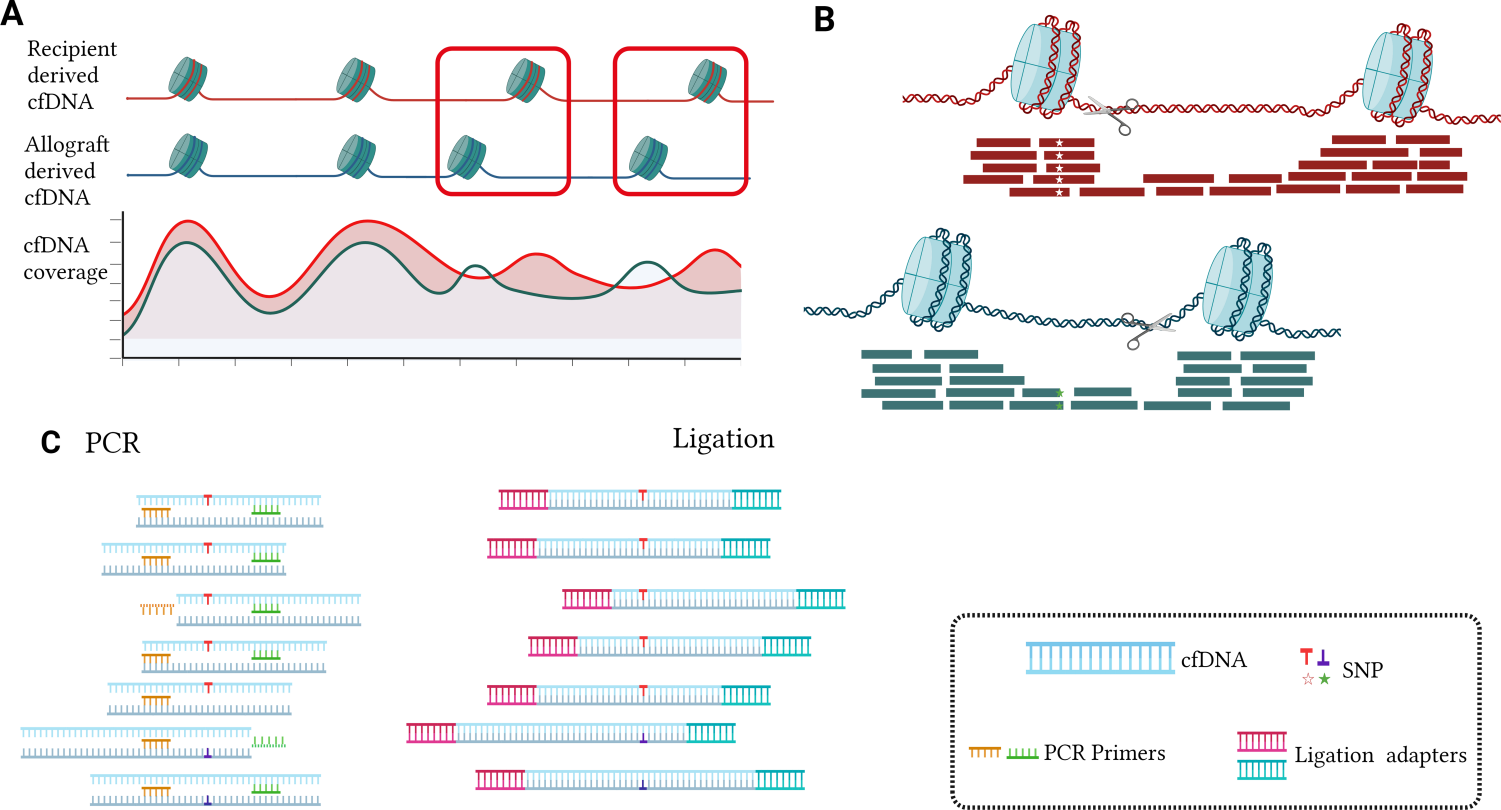
The impact of nucleosome footprinting and detection methods on targeted selected SNPs **(A, B)**. In the nucleosome-wrapped regions, there is a higher coverage of cfDNA. Additionally, at certain positions in the genome, there exist differences in the distribution of nucleosomes between the donor and recipient in organ transplantation **(A)**. Nucleosome cutting typically occurs outside the nucleosome-wrapped regions, resulting in cfDNA fragments carrying the same SNP exhibiting different fragmentation patterns. When the SNP is located within the recipient’s nucleosome wrapping region rather than the donor’s nucleosome wrapping region, it may lead to a decrease in the donor SNP signal, causing ddcfDNA detection values to be lower than the actual values **(B)**. When the SNP is biased towards one end of the cfDNA fragment, the conventional PCR approach tends to overlook specific cfDNAs bearing SNPs because they require both forward and reverse primers to bind simultaneously for double-stranded amplification, leading to an underestimation of the target quantity. However, Ligation PCR facilitates the capture of these cfDNA fragments by adding PCR primer adapters to the cfDNA. This ensures that conventional PCR primers can easily capture these cfDNA fragments without being biased towards a specific end due to SNP presence, thereby increasing the accuracy of detection **(C)**.

Nucleosomes, which are fundamental units of DNA packaging in eukaryotes, vary in positioning across organs (20, 21). This results in heterogeneity of endonuclease cleavage reactions in donor and recipient genomes and PCR primer binding bias during amplification (**Figure 2**). Therefore, it is advisable to avoid selecting SNPs on nucleosome footprinting specifically in kidney tissue when using PCR platforms. Furthermore, when utilizing ligation technology, SNPs can be detected regardless of cfDNA fragment endpoints, thus decreasing nucleosome bias (**Figure 2**). Moreover, the influence of nucleosome footprints is insignificant in circulating tumor DNA and cell-free fetal DNA (cffDNA) examinations as the focus is mainly on qualitative assessment (22, 23).

### 2.3 How many target SNPs/Indels are enough?

Determining the optimal number of target SNPs/Indels is crucial. Research through the NGS platform uses hundreds or more SNPs. For example, Grskovic et al. (16) used 266 SNPs, Sigdel et al. used 13392 SNPs (24), while Zhou et al. used 5800 SNPs (18). In a study involving whole genome sequencing, 640 – 700K SNPs were used (13), while research using the ddPCR technology platform targeted dozens of SNPs (25), and qPCR detection also used only a few indel polymorphisms loci (26). A larger number is advantageous, but cost constraints demand a minimum. In scenarios where donor genotype info is unavailable, obtaining at least two LFHS (A/a and a/a) simultaneously is necessary. So, what is the appropriate number of SNPs?

For the calculation, assuming *m* as the population’s probability of the “A” genotype and 1-*m* for “a” genotype, three scenarios can occur:

1) $\;P1\;=\;P\;(R\;=\;AA,\;D\;=\;aa\;or\;R\;=\;aa,D\;=\;AA)\;=\;2\;\times \;m^{2}\;\times \;{\left(1-m\right)}^{2}$

2) $\;P2\;=\;P(R\;=\;AA,\;D\;=\;Aa\;or\;R\;=\;aa,\;D\;=\;Aa)\;=\;2m\;-\;6m^{2}\;+\;8m^{3}\;-\;4m^{4}$

3)  P3 = 1 − P1 − P2

The expected number of SNPs for at least one occurrence of scenario 1 (L_1,0,0_) can be calculated as Eq. 1, considering: 1) There is a probability (*P*1) of scenario 1 happening in the current SNP; 2) There is a probability (*P*2 + *P*3) of either scenario 2 or 3 happening in the current SNP, and in that case, starting over with one more SNP (1 +*L*_1,0,0_). By solving this equation, *L*_1,0,0_ = 1/*P*1. Similarly, *L*_1,0,0_ = *i*/*P*1, and *L*_0,_ *_j_*_,0_ = *j/P*2.


(1) $$L_{1,0,0}\;=\;P1\times 1\;+\;\left(P2\;+\;P3\right)\times \left(1\;+\;L_{1,0,0}\right)$$

Similarly, the expected number of SNPs necessary for scenario 1 to happen *i* times and scenario 2 to occur *j* times (*L*_i,j,0_) can be calculated using Equation 2:


(2) $$\begin{array}{c} L_{i,j,0}\;=\;P1\times \left(L_{i-1,j,0}\;+\;1\right)+\;P2\times \left(L_{i,j-1,0}\;+\;1\right)+\;P3\times \left(L_{i,j,0}+\;1\right)\\ \Leftrightarrow L_{i,j,0}=\frac{1+P1\times L_{i-1,j,0}+P2\times L_{i,j-1,0}}{P1+P2} \end{array}$$

Practically, for m = 0.4, *L*_1,1,0_ is approximately 10, and *L*_1,2,0_ is approximately 13. This indicates that on average, about 13 SNPs need to be observed for one instance of “A/a” and two instances of “a/a” to occur. The above calculations are carried out on the assumption that there is no relationship between the donor and the recipient.

### 2.4 How many sequencing depths are needed?

Sequencing depth is crucial for %ddcfDNA detection. For one SNP (homozygous recipients, homozygous donors, and with different genotypes), the actual %ddcfDNA of *p*, *y* follows a binomial distribution which refers to the number of reads observed, LFHS, at depth *n*. Considering that the probability of the observed %ddcfDNA (*p*’ = *y*/*n*) falling within a relative error range of the actual %ddcfDNA should not be less than 90%, *n* is calculated by Equation 3.


(3) $$\begin{array}{c} P(p\left(1-re\right)<p^{\prime }<p\left(1+re\right))\geq 0.9\\ \Leftrightarrow P\left(\frac{p\left(1-re\right)-p}{\sqrt{\frac{p\left(1-p\right)}{n}}}<\frac{\frac{y}{n}-p}{\sqrt{\frac{p\left(1-p\right)}{n}}}<\frac{p\left(1+re\right)-p}{\sqrt{\frac{p\left(1-p\right)}{n}}}\right)\geq 0.9\\ \Leftrightarrow \frac{\text{p}\left(1+\text{re}\right)-\text{p}}{\sqrt{\frac{\text{p}\left(1-\text{p}\right)}{\text{n}}}}>1.645\;\\ \Leftrightarrow n>{\left(\frac{1.645}{re}\right)}^{2}\times \frac{1-p}{p} \end{array}$$

**Figure 3:**
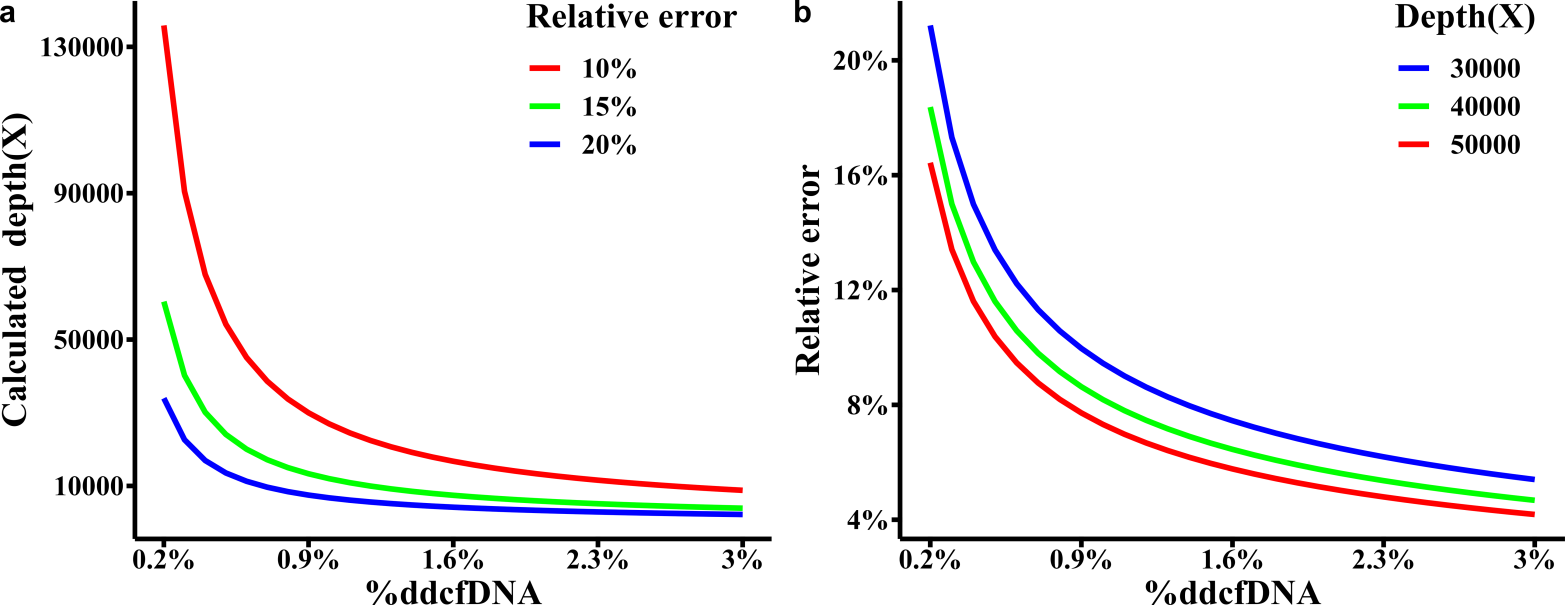
**(A)** Minimum required sequencing depth and **(B)** relative errors for %ddcfDNA.

**Figure 3A** specifies the necessary sequencing depth for various %ddcfDNA (ranging from 0.2% to 3%) with a relative error. Notably, when the actual %ddcfDNA is 0.2%, a minimum sequencing depth of 135,853X is needed with a relative error of 10%. As the actual %ddcfDNA increases, the minimum required sequencing depth gradually decreases. For instance, at %ddcfDNA of 3%, only 8,803X is required.

In kidney transplantation, %ddcfDNA diagnostic threshold is 1%. Therefore, it is desirable for the actual concentration to range between 0.2% and 1.5% (considering an absolute error of 0.001), resulting in a depth range of 5,435X to 40,225X. Similarly, for an expected %ddcfDNA of 1.5% to 3% (considering an absolute error of 0.005), the depth range is 1,609X to 3,169X. Thus, under the assumption of a homozygous donor and recipient with differing genotypes at the known locus, a reasonable sequencing depth should be around 50,000X. **Figure 3B** illustrates the relative errors at various %ddcfDNA within a 90% confidence interval when the sequencing depth is set at 50,000X. Whereas, the relative error rate for ddPCR is around 35-40% when the %ddcfDNA is 0.5% (27). Moreover, in diagnosing TCMR, newer reports are employing diagnostic thresholds lower than 1%, indicating that we may actually need higher sequencing depth.

It is worth noting that when hybrid capture sequencing depth reaches high, the presence of a certain proportion of off-target reads can result in high costs. In such cases, the sequencing cost can be reduced by increasing the number of SNPs. In binomial distribution, 50,000X depth at one SNP equals to 2,000X at 25 SNPs. As mentioned in Section 2.3, *L*_25,50,0_ is around 231. Thus, 231 SNPs with the average depth of 2,000X is recommended. With this approach, it should identify effective SNPs where the recipient is homozygous, the donor is homozygous, and their genotypes are different among the 231 SNPs. Utilizing the sum of small signal reads across all effective SNPs divided by the total sequencing depth at these SNPs, it is possible to estimate the %ddcfDNA. This estimation provides a 90% confidence interval for relative error ranging from 0.04 to 0.017.

Second, the effective sequencing depth is also related to the amount of cfDNA extraction (28, 29). If the total extraction amount of cfDNA is 20 ng/mL plasma, on average 1 copy of genome equivalent is about 6.6 pg, then 20 ng cfDNA will be equal to 3030 genome equivalents. Hence, the highest effective sequencing depth is 3030X. Since the cfDNA extraction volume can impact low-frequency heterogeneous SNPs detection, it is necessary to ensure the extraction of optimal cfDNA amount in heart transplantation because the cfDNA derived from stable allograft is usually less than 0.2% (30). For kidney transplant recipients, studies have reported a median ddcfDNA of 0.33% for non-rejection group as diagnosed via molecular sign-out (31). In order to detect small signals from donor alleles on informative SNP loci, the detection accuracy needs to reach 0.165%. Considering that hybridization capture methods typically exhibit a coefficient of variation of 25% (32), and assuming the coverage of SNP loci follows a normal distribution, a minimum cfDNA amount of 8 ng is required in kidney transplant to ensure that 95% of SNPs meet the detection criteria.

## 3 Fraction and absolute values of ddcfDNA

Approximately 80% of plasma cfDNA is derived from the apoptosis and necrosis of white blood cells (WBC), commonly referred to as leukocyte-derived cfDNA. The next highest fraction comes from liver, with the remaining cfDNA originating from various organs in the body (33, 34).

The fraction value of ddcfDNA refers to the proportion of ddcfDNA in relation to the total cfDNA (Eq. 4). It is relatively less influenced by internal experimental factors, such as cfDNA preservation and extraction steps. The diagnostic utility of ddcfDNA fraction values in the context of renal allograft rejection has been extensively validated (35). The absolute value of ddcfDNA represents the amount of ddcfDNA per millilitre of plasma or urine (expressed in ng or genome copies, Equation 5) (36). The calculation formulae are as follows:


(4) $$\%ddcfDNA=\frac{Q\;donor}{Q\;donor+Q\;recipient}\times 100$$

(5) $$ddcfDNA\;\left(\frac{ng}{mL}\;or\frac{copies}{mL}\right)=ddcfDNA\%\times cfDNA\;extraction\;volume$$

Several studies have suggested that the absolute value of ddcfDNA is of greater diagnostic significance for rejection (25, 37). It is speculated that in various clinical conditions characterized by minor fluctuations in leukocyte-derived cfDNA, the absolute value of ddcfDNA may be less influenced by factors such as sepsis, urinary tract infection, methylprednisolone pulse therapy, degree of renal tubular and interstitial inflammation, including vigorous physical activity, among others. Both fraction and absolute values have their respective advantages and disadvantages (**Table 1**). The efficacy of ddcfDNA fraction and absolute values is investigated in different pathological conditions.

**Table 1:** Comparison of advantages and disadvantages between absolute and fraction values.

Metric	Advantages	Disadvantages
Absolute value	1) Not influenced by fluctuations in WBC-derived cfDNA levels; 2) Not affected by variations in the extent of inflammation in different pathological conditions; 3) Not influenced by recipient cell lysis during blood drawn.	1) Poor comparability between different experimental platforms (plasma/urine cfDNA preservation devices, extraction kits, etc.); 2) Long-term storage of plasma/urine samples, even on the same experimental platform, can also impact results.
Fraction value	1) Not influenced by total cfDNA degradation and extraction reagents; 2) Strong comparability between different experimental platforms.	1) Prone to fluctuations in WBC levels, such as in the case of rejection detection during infection states.

### 3.1 TCMR, ABMR and BKVN

The value of ddcfDNA in the diagnosis of ABMR has been recognized by researchers (38), while the value of ddcfDNA in the diagnosis of T-cell-mediated rejection (TCMR) is currently controversial. Some studies show little significance (35), while others report significance, especially in the case of absolute values. Oellerich’s research suggests that ddcfDNA can effectively diagnose rejection, including TCMR and ABMR. When diagnosing borderline TCMR, the significance p-value of ddcfDNA absolute values (*p* < 0.0001) is much better than fraction values (0.0006) (25). Bunnapradist et al. found that combining the absolute and fraction values of ddcfDNA improved the diagnosis of rejection. Specifically, introducing an absolute ddcfDNA threshold (≥78 copies/mL) alongside the fraction threshold (≥ 1%) corrected false-negative results in 2 cases of TCMR (39).

In Bunnapradist et al.*’s* study, it was found that the proportion of TCMR (excluding mixed rejection) accounts for 83.3% of the active rejection group (39). However, according to Gohh’s research, a high absolute value does not provide any further discriminatory power over ddcfDNA fraction in detecting allograft rejection, with TCMR (excluding mixed rejection) accounting for only 60% (40). TCMR is primarily associated with T-cell inflammation, and the apoptosis and necrosis of inflammatory cells could increase leukocyte-derived cfDNA, which would lead to lower fraction values (1). Therefore, the absolute values of ddcfDNA appear to have greater significance in diagnosing TCMR compared to fraction values. However, due to differences in cohort selection between these two studies, the former employed a retrospective for-cause ddcfDNA test, while the latter utilized a prospective for-surveillance ddcfDNA test, with variation in sample sizes between them. Therefore, further research is needed to assess the diagnostic value of absolute values in TCMR.

Chen et al. documented seven cases of probable BK virus nephropathy (BKVN), defined by the presence of a sustained plasma BKV DNA load >10^3^ copies/mL in two measurements within three weeks, coupled with negative anti-SV40-T IHC staining on renal biopsy. These 7 patients had a high urinary %ddcfDNA (mean value = 91%), and their absolute values were not significantly different from the proved-BKVN group but showed a significant difference compared to the resolving group (11).

It was hypothesized that these 7 patients were most likely in the early stages of BKVN with a low degree of tubulitis and interstitial inflammation. According to another study, the BKVN-pure group, which indicates patients with proven BKVN who had no histological evidence of rejection according to the Banff 2017 criteria, also had a higher fraction value than the BKVN-proved and TCMR groups, but the absolute value was not higher than the BKVN-proved group. This BKVN-pure group also shows mild tubulitis (41).

Similarly, Whitlam et al. reported an increased fraction value of ddcfDNA in patients with chronic active ABMR (caABMR), while the absolute value was low (37). This suggests that under the pathological condition of caABMR, although there is rejection injury, the degree of inflammation is mild.

Overall, when the degree of inflammation is low, the fraction value will be higher (Equation 4). Meanwhile, when the degree of inflammation is greater, the WBC will release more cfDNA, and the amount of donor source will be further diluted, and the fraction value will be low (Equation 4).

### 3.2 Infection

#### 3.2.1 Lung infection

Lung infections can elevate WBC levels, resulting in an increase of leukocyte-derived cfDNA in plasma (42, 43). Consequently, infection dramatically increases the total plasma cfDNA extracted and increases the “Q recipient” value in Eq.4, causing a decrease in the fraction value of ddcfDNA. However, the absolute value of ddcfDNA remains unaffected. When the fraction value of ddcfDNA drops to the limit of detection (e.g., 0.1% - 0.2% for NGS), the Q recipient value increases, but the fraction value of ddcfDNA no longer decreases. Therefore, calculating the absolute value using the fraction value of ddcfDNA is not feasible at this time.

In a study, thirteen patients with sepsis were enrolled and their plasma ddcfDNA was measured. It was found that the fraction value of ddcfDNA at the onset of sepsis was not different from that after treatment and in stable state, but the absolute value was increased, indicating that kidney damage was still present in sepsis (36).

Another study found that three patients who experienced rejection were also infected with pathogens and their %ddcfDNA was below the cut-off value (44). Jose et al. found that renal transplant patients infected with COVID-19 had elevated levels of total plasma cfDNA (7.9 multiples of median [MoM]). Two patients with biopsy-confirmed acute cellular rejection had ddcfDNA fractions below the 1% cut-off for rejection (0.20% and 0.78%), but the total cfDNA levels elevated to 7.9 MoM and 4.8 MoM, respectively (45).

#### 3.2.2 Urinary tract infection

Urinary tract infections can lead to an increase in recipient immune cells in the urinary tract following immune activation, resulting in increased levels of cfDNA derived from WBC (46). Recipients with pyuria had a lower urinary ddcfDNA fraction than no-pyuria (*p* = 8.0 × 10^−4^) (47). Patients with tubulitis and interstitial inflammation also show immune cell activation.

#### 3.2.3 B19 virus infection

The fraction value of ddcfDNA in patients with B19 virus infection was lower than the reported cut-off value for rejection. However, its absolute value was significantly higher than the reported cut-off value (data to be published). The B19 virus primarily infects precursor of red blood cells (nucleated cells) where it induces cell cycle arrest in the G1 phase through NS1, leading to apoptosis and damage of the precursor of red blood cells (48, 49), and subsequent increase in the release of total cfDNA.

In summary, both absolute and fractional values need to be evaluated in the detection of ddcfDNA, and different combinations of absolute and fractional values represent different meanings.

## 4 Effects of different sampling time points

### 4.1 Effects of early ischemia-reperfusion after transplantation

Studies have shown that early postoperative ischemia-reperfusion injury (IRI) increases the detection value of ddcfDNA, which gradually decreases to less than 1% by about the 7^th^ day after surgery (8). Studies of ddcfDNA levels in early postoperative rejection patients showed that there was no significant difference in ddcfDNA levels between the rejection and non-rejection groups within 10 days of surgery. Between 11 and 180 days after surgery, ddcfDNA levels were significantly higher in the rejection group than that in the non-rejection group (*p* < 0.05) (50). When using ddcfDNA testing to diagnose the risk of rejection, it is important to avoid samples collected within the first 10 days after transplantation. This limitation indicates that ddcfDNA may not be effective in detecting rejection shortly after transplantation.

### 4.2 Effects of deletional induction or rejection therapy

Studies have reported a rapid decrease in %ddcfDNA in both TCMR and ABMR patients after treatment for rejection. It has been suggested that ddcfDNA could serve as a potential biomarker for real-time monitoring of response to therapy (51). Shen et al. found that %ddcfDNA decreased within 3 days post injection of methylprednisolone sodium succinate in 28 patients with acute renal rejection (from 2.566% to 0.773%, *p* < 0.001) (10). The decrease in ddcfDNA is thought to be non-specific as the half-life of cfDNA is very short (16 min - 2 h). It should be noted that there is a rapid decrease in inflammatory cells after steroid pulse therapy, especially methylprednisolone pulses, which may also contribute to the decrease in ddcfDNA. It was thought that recipients treated with deletional induction or rejection therapies (multi-doses of Thymoglobulin or long lasting alemtuzumab) may impact overall leukocyte numbers and cfDNA fraction calculations. Therefore, it is advisable to obtain samples prior to treatment for rejection or during the stable period after treatment when utilizing ddcfDNA.

### 4.3 Effects of renal biopsy

Whitlam et al. found that there were no statistically significant differences in the distribution of ddcfDNA absolute value (*p* = 0.19), total cfDNA (*p* = 0.55), and ddcfDNA fraction value (*p* = 0.7) measurements between before and after renal biopsy (37). Here “before” refers to the time before the puncture, “after” refers to the day after the puncture, since the half-life of cfDNA is very short, this interval is relatively long, and the results may not reflect the effect of renal biopsy on ddcfDNA. Kyeso et al. found that the ddcfDNA levels increased at 20 min (*p* = 0.0022) and 2h (*p* = 0.0138) after biopsy in 16 renal transplant patients and there was no significant change at 24 – 48 h after biopsy (*p* = 0.2846) (9). Further research can be further subdivided into 2 - 24 h, such as 2 - 6 and 6 - 24 h after surgery.

### 4.4 Effects of strenuous exercise and high BMI

Exercise can induce the formation of neutrophil extracellular traps (NETs). After NET rupture, cfDNA in NETs is released into the extracellular space. One study showed that the amount of cfDNA could increase significantly (from 3.3 ng/mL to 28.9 ng/mL, *p* = 0.002) during exhaustive exercise (at a speed corresponding to 70% of their personal VO_2_max) (52). The amount of plasma cfDNA was also significantly increased to 7.0-fold (*p* < 0.001) in the incremental exercise state (53), and cfDNA was also significantly increased after high-intensity interval exercise (*p* = 0.047). Therefore, it is important to maintain the resting state as much as possible before blood collection, e.g. it may be advisable for the patient to have complete bed rest after climbing stairs.

Obesity may also increase the release of total cfDNA, possibly due to the generation of free radicals and oxidative stress, which are associated with cellular and molecular damage in cell injury (54). Among patients with a BMI ≥ 24 kg/m², there is a significant increase in total cfDNA levels compared to those with a BMI < 24 kg/m² (*p* = 0.0008) (55). The increase in total cfDNA may dilute ddcfDNA, but the specific magnitude of this effect is still unknown. Research on the fetal fraction of cell-free DNA (ffcfDNA) in the plasma of obese pregnant women suggests that the decrease in ffcfDNA may be due to obesity being associated with reduced release of cfDNA from the placenta. Further research is needed to fully understand the impact of obesity on ddcfDNA.

## 5 Impact of confounding signals

The current technology for detecting ddcfDNA is primarily a “donor-independent method”. When using NGS detection technology, there are two important considerations: 1) The donor-independent technique assumes that the signal ratio of the donor organ is less than 15-20% (19). If the actual signal from donor is above this threshold, it can lead to biased quantification when the donor genotype is independent, therefore liver transplantation must simultaneously genotype the leukocyte DNA of the recipient. 2) It is difficult to distinguish the individual kidney signal when there are other confounding signals, especially those with signal values higher than the background noise of sequencing.

### 5.1 Multi-organ transplantation

In renal transplant patients with combined organ transplantation, the donor signal has two sources: the donor kidney and other combined organs. Because most of the time, the joint organ comes from the same donor, and their genomes are the same, it is difficult to distinguish the individual signal sources of the donor kidney via SNP typing.

In cases of simultaneous pancreas-kidney transplantation, 40% of patients with rejection will have concurrent rejection. If kidney biopsy is performed alone, 26.5% of the pancreas rejections may be missed (56), but pancreatic rejection is difficult to diagnose in terms of pathological or serological indicators. Thus, ddcfDNA can be used as a non-invasive marker for detecting pancreatic rejection. In a study of ddcfDNA on simultaneous pancreas-kidney transplantation, it was found that the ddcfDNA value at one-month post-transplantation was higher than KT alone (0.9 ± 1.1% VS 0.49 ± 0.4%) (57). Baseline ddcfDNA levels among normal PTx (25 SPKT and 3 PT alone) recipients show a median level of <1.0% (similar to KT alone), but a higher mean within one-month post-transplantation (1.00 ± 0.9%) (58). In the condition of rejection, Ventura-Aguiar et al. discovered the fraction value (0.83% versus 0.30%; *p* = 0.006) and absolute value (81.3 versus 35.3 cp/mL; *p* = 0.001) of ddcfDNA were elevated compared to the stable group after 45-day posttransplant. However, as it is not possible to differentiate the signal source from which donor organ specifically, determining whether it is a case of kidney or pancreas rejection remains unfeasible (59).

For other combined kidney transplants, such as combined liver-kidney transplants, liver-derived cfDNA accounts for more than 10% (60, 61), while the level of cfDNA from donor kidney is low (~ 1%), and the cfDNA from donor kidney accounts for about 1/10 of the transplanted liver. Therefore, in combined liver-kidney transplantation, the diagnosis of transplanted liver rejection is not affected (cutoff value is 10%), but the diagnosis of transplanted kidney rejection is very difficult. At this time, conducting tissue-specific cfDNA methylation sequencing is a potential avenue for future exploration.

### 5.2 Dual kidney transplantation

Dual kidney transplantation (DKT) allows for the use of marginal kidneys that are not suitable for single kidney transplantation. The amount of cfDNA, which is derived from apoptotic cells of transplanted kidneys, is theoretically higher in DKT compared to single kidney transplantation. A study on median dd-cfDNA levels at one-month post-surgery showed that patients undergoing dual kidney transplantation (1.10%, n = 3) had higher median dd-cfDNA levels compared to patients undergoing single kidney transplantation (0.31%), but the difference exhibited no statistical significance (62). Thus, dual kidney transplants may contribute to increased basic levels of ddcfDNA.

### 5.3 Renal transplant patients with tumors, bone marrow transplantation, blood transfusion treatment or in gestational period

Tumors, pregnant women, bone marrow transplantation and blood transfusion will all introduce heterologous cfDNA (63, 64). Currently, NGS-based ddcfDNA detection methods are employed in kidney transplantation, of which most of them are donor genotype-independent (16, 18, 24), and directly calculates low heterologous signals to obtain the proportion of donors. Therefore, factors that affect the proportion of heterologous signals will affect the fraction of ddcfDNA. If the patient’s condition is unclear, the presence of the other type of heterologous signal can be evaluated from several aspects: 1) How many informative SNPs can be detected? According to Mendel’s laws, when the donor and the recipient are unrelated, the number of informative SNPs is theoretically accounting for 37.5% of the total SNP. When the donor and recipient are siblings or parent/child, the informative SNPs are accounting for 21.875% and 25%. 2) In addition, there may also be abnormalities in quantitative value, e.g kidney transplantation recipient diagnosed with liver cancer will exhibit chromosome copy variation (CNV) on haploids in tumor cells which can disrupt the law of genotype distribution frequency. This can result in higher ddcfDNA values, since cfDNA derived from liver cancer cells often exceeds 15% in blood. In our empirical observations, patients diagnosed with liver cancer often displays a plasma ddcfDNA test value of 20%, significantly higher than that observed among kidney injury patients. Under these conditions, it is viable to increase sequencing depth and isolate the two signal sources, that is, the heterologous signal proportions between the donor kidney and the liver cancer, which may manifest as two ddcfDNA value cluster.

## 6 Outlook

ddcfDNA shows promise as a biomarker for assessing renal allograft injury, but further considerations must be taken into account when utilising ddcfDNA testing.

1. The current high-throughput sequencing technology allows for donor-genotype-independent quantification and convenient operation. Retesting patients no longer requires specific PCR primers and probes for each patient, while also eliminating the impact of nucleosome mapping on the heterologous signal bias at specific SNP sites. To reduce background noise in tests, such as the ddcfDNA test for heart transplant patients, the polymorphic Indel site on the human genome could be used in the future. Unlike SNP, it is less susceptible to sequencing errors and can serve as a new biomarker. It is recommended to draw over 10 mL of blood for cfDNA extraction to improve the identification of LFHS and ensure adequate absolute copy number detection.
2. The detection of renal allograft injury is improved by considering both the absolute and fraction values of ddcfDNA. Therefore, interpretation of ddcfDNA results requires evaluation of both parameters. However, it is crucial to recognize that absolute values of ddcfDNA are not readily comparable across different laboratories. Furthermore, on the same ddcfDNA testing platforms, comparing absolute values obtained from samples with different cryopreservation times is also challenging due to the degradation of cfDNA during prolonged cryopreservation (65–67). Additionally, it should be considered that the amount of cfDNA extraction may be affected by using different blood preservation tubes (68).
3. The diagnostic value of plasma and urine ddcfDNA for transplanted kidney injury varies. Plasma ddcfDNA levels are mainly associated with glomerulonephritis and PTC scores in Banff scores (7). However, in cases of TCMR or BKVN, the primary indication of injury is renal tubular and interstitial damage (Banff 2019). Urine DNA detection methods are becoming increasingly matured (69), and it may be advantageous to measure ddcfDNA levels in both plasma and urine when detecting both rejection and BKVN risk simultaneously (6, 41). If a renal transplant patient is complicated by tumor, bone marrow transplantation/blood transfusion therapy, or pregnancy, detection through SNP is more challenging due to the signal of a confounding second heterologous DNA. In such cases, tissue-specific methylation detection may be required.
4. One of the main challenges confronting organ transplantation, aside rejection and post-transplant infection, is the availability of suitable donors. Increasing donor pool to clear the backlog of recipients on the waiting list has been of interest to physicians. Transplant using organs from ‘foreign’ species is therefore becoming a common practice due to advances in heterogenous donor-recipient compatibility even though rejection and zoonotic infection remain a challenge in this field. The potential of circulating xenograft derived cfDNA as a marker of rejection is least explored and should be investigated as a safe, effective and non-invasive method for continuous monitoring of graft status in xenotransplantation.

## References

[1] Zhou Y Cheng D Jiang T. The role of donor-derived cell-free DNA in the detection of renal allograft injury. Nephrologie Therapeutique. (2021) 17:12–7. doi: 10.1016/j.nephro.2020.10.003 33454228

[2] Oellerich M Sherwood K Keown P Schütz E Beck J Stegbauer J. Liquid biopsies: donor-derived cell-free DNA for the detection of kidney allograft injury. Nat Rev Nephrol. (2021) 17:591–603. doi: 10.1038/s41581-021-00428-0 34031575

[3] Aubert O Ursule-Dufait C Brousse R Gueguen J Racapé M Raynaud M. Cell-Free DNA for the detection of kidney allograft rejection. Nat Med. (2024) 30:2320–7. doi: 10.1038/s41591-024-03087-3 PMC11333280 38824959

[4] Gielis EM Beirnaert C Dendooven A Meysman P Laukens K De Schrijver J. Plasma donor-derived cell-free DNA kinetics after kidney transplantation using a single tube multiplex PCR assay. PloS One. (2018) 13:e0208207. doi: 10.1371/journal.pone.0208207 30521549 PMC6283554

[5] Cheng D Liu F Xie K Zeng C Li X Ni X. Donor-derived cell-free DNA: An independent biomarker in kidney transplant patients with antibody-mediated rejection. Transpl Immunol. (2021) 69:101404. doi: 10.1016/j.trim.2021.101404 33971294

[6] Chen XT Qiu J Wu ZX Zhang H Chen T Yang SC. Using both plasma and urine donor-derived cell-free DNA to identify various renal allograft injuries. Clin Chem. (2022) 68:814–25. doi: 10.1093/clinchem/hvac053 35587713

[7] Guo L Shen J Lei W Yan P Wang M Zhou Q. Plasma donor-derived cell-free DNA levels are associated with the inflammatory burden and macrophage extracellular trap activity in renal allografts. Front Immunol. (2022) 13:796326. doi: 10.3389/fimmu.2022.796326 35386710 PMC8977515

[8] Shen J Zhou Y Chen Y Li X Lei W Ge J. Dynamics of early post-operative plasma ddcfDNA levels in kidney transplantation: a single-center pilot study. Transplant Int. (2019) 32:184–92. doi: 10.1111/tri.13341 30198148

[9] Kyeso Y Bhalla A Smith AP Jia Y Alakhdhair S Ogir SC. Donor-derived cell-free DNA kinetics post-kidney transplant biopsy. Transplant Direct. (2021) 7:E703. doi: 10.1097/TXD.0000000000001149 34056078 PMC8154469

[10] Shen J Guo L Yan P Zhou J Zhou Q Lei W. Prognostic value of the donor-derived cell-free DNA assay in acute renal rejection therapy: A prospective cohort study. Clin Transplant. (2020) 34:e14053. doi: 10.1111/ctr.14053 32735352

[11] Chen XT Chen WF Li J Deng RH Huang Y Yang SC. Urine donor–derived cell-free DNA helps discriminate BK polyomavirus-associated nephropathy in kidney transplant recipients with BK polyomavirus infection. Front Immunol. (2020) 11:1763. doi: 10.3389/fimmu.2020.01763 32973745 PMC7466716

[12] De Vlaminck I Valantine HA Snyder TM Strehl C Cohen G Luikart H. Circulating cell-free DNA enables noninvasive diagnosis of heart transplant rejection. Sci Transl Med. (2014) 6:241ra77. doi: 10.1126/scitranslmed.3007803 PMC4326260 24944192

[13] Sharon E Shi H Kharbanda S Koh W Martin LR Khush KK. Quantification of transplant-derived circulating cell-free DNA in absence of a donor genotype. PloS Comput Biol. (2017) 13:e1005629. doi: 10.1371/journal.pcbi.1005629 28771616 PMC5542400

[14] Park S Guo K Heilman RL Poggio ED Taber DJ Marsh CL. Combining blood gene expression and cellfree dna to diagnose subclinical rejection in kidney transplant recipients. Clin J Am Soc Nephrol. (2021) 16:1539–51. doi: 10.2215/CJN.05530421 PMC8499014 34620649

[15] Beck J Oellerich M Schütz E. A universal droplet digital PCR approach for monitoring of graft health after transplantation using a preselected SNP set. In: Karlin-Neumann G Bizouarn F, editors. Digital PCR: methods and protocols. Springer New York, New York, NY (2018). p. 335–48. doi: 10.1007/978-1-4939-7778-9_19 29717452

[16] Grskovic M Hiller DJ Eubank LA Sninsky JJ Christopherson C Collins JP. Validation of a clinical-grade assay to measure donor-derived cell-free DNA in solid organ transplant recipients. J Mol Diagnostics. (2016) 18:890–902. doi: 10.1016/j.jmoldx.2016.07.003 27727019

[17] Altuğ Y Liang N Ram R Ravi H Ahmed E Brevnov M. Analytical validation of a single-nucleotide polymorphism-based donor-derived cell-free DNA assay for detecting rejection in kidney transplant patients. Transplantation. (2019) 103:2657–65. doi: 10.1097/TP.0000000000002665 PMC6867667 30801536

[18] Zhou Y Yang G Liu H Chen Y Li X Ge J. A noninvasive and donor-independent method simultaneously monitors rejection and infection in patients with organ transplant. Transplant Proc. (2019) 51:1699–705. doi: 10.1016/j.transproceed.2019.04.051 31399160

[19] McKenna A Hanna M Banks E Sivachenko A Cibulskis K Kernytsky A. The genome analysis toolkit: A MapReduce framework for analyzing next-generation DNA sequencing data. Genome Res. (2010) 20:1297–303. doi: 10.1101/gr.107524.110 PMC2928508 20644199

[20] Ulz P Thallinger GG Auer M Graf R Kashofer K Jahn SW. Inferring expressed genes by whole-genome sequencing of plasma DNA. Nat Genet. (2016) 48:1273–8. doi: 10.1038/ng.3648 27571261

[21] Snyder MW Kircher M Hill AJ Daza RM Shendure J. Cell-free DNA comprises an in vivo nucleosome footprint that informs its tissues-of-origin. Cell. (2016) 164:57–68. doi: 10.1016/j.cell.2015.11.050 26771485 PMC4715266

[22] Zhang M Li K Qu S Guo Z Wang Y Yang X. Integrative analyses of maternal plasma cell-free DNA nucleosome footprint differences reveal chromosomal aneuploidy fetuses gene expression profile. J Transl Med. (2022) 20:536. doi: 10.1186/s12967-022-03735-7 36401256 PMC9673457

[23] Vanderstichele A Busschaert P Landolfo C Olbrecht S Coosemans A Froyman W. Nucleosome footprinting in plasma cell-free DNA for the pre-surgical diagnosis of ovarian cancer. NPJ Genom Med. (2022) 7:30. doi: 10.1038/s41525-022-00300-5 35484288 PMC9050708

[24] Sigdel TK Archila FA Constantin T Prins SA Liberto J Damm I. Optimizing detection of kidney transplant injury by assessment of donor-derived cell-free dna via massively multiplex pcr. J Clin Med. (2019) 8:19. doi: 10.3390/jcm8010019 PMC6352163 30583588

[25] Oellerich M Shipkova M Asendorf T Walson PD Schauerte V Mettenmeyer N. Absolute quantification of donor-derived cell-free DNA as a marker of rejection and graft injury in kidney transplantation: Results from a prospective observational study. Am J Transplant. (2019) 19:3087–99. doi: 10.1111/ajt.15416 PMC6899936 31062511

[26] Dauber EM Kollmann D Kozakowski N Rasoul-Rockenschaub S Soliman T Berlakovich GA. Quantitative PCR of INDELs to measure donor-derived cell-free DNA—a potential method to detect acute rejection in kidney transplantation: a pilot study. Transplant Int. (2020) 33:298–309. doi: 10.1111/tri.13554 PMC7065216 31710731

[27] Deprez L Corbisier P Kortekaas AM Mazoua S Beaz Hidalgo R Trapmann S. Validation of a digital PCR method for quantification of DNA copy number concentrations by using a certified reference material. Biomol Detect Quantif. (2016) 9:29–39. doi: 10.1016/j.bdq.2016.08.002 27617230 PMC5007884

[28] Yuan Z Wang X Geng X Li Y Mu J Tan F. Liquid biopsy for esophageal cancer: Is detection of circulating cell-free DNA as a biomarker feasible? Cancer Commun. (2021) 41:3–15. doi: 10.1002/cac2.12118 PMC7819547 33264481

[29] Keppens C Dequeker EMC Patton SJ Normanno N Fenizia F Butler R. International pilot external quality assessment scheme for analysis and reporting of circulating tumour DNA. BMC Cancer. (2018) 18:804. doi: 10.1186/s12885-018-4694-x 30092778 PMC6085634

[30] Agbor-Enoh S Shah P Tunc I Hsu S Russell S Feller E. Cell-free DNA to detect heart allograft acute rejection. Circulation. (2021) 143:1184–97. doi: 10.1161/CIRCULATIONAHA.120.049098 PMC8221834 33435695

[31] Halloran PF Reeve J Madill-Thomsen KS Demko Z Prewett A Billings P. The trifecta study: comparing plasma levels of donor-derived cell-free DNA with the molecular phenotype of kidney transplant biopsies. J Am Soc Nephrol. (2022) 33:387–400. doi: 10.1681/ASN.2021091191 35058354 PMC8819982

[32] Feng Y Chen D Wang GL Zhang VW Wong LJC. Improved molecular diagnosis by the detection of exonic deletions with target gene capture and deep sequencing. Genet Med. (2015) 17:99–107. doi: 10.1038/gim.2014.80 25032985 PMC4338802

[33] Sun K Jiang P Chan KCA Wong J Cheng YKY Liang RHS. Plasma DNA tissue mapping by genome-wide methylation sequencing for noninvasive prenatal, cancer, and transplantation assessments. Proc Natl Acad Sci U.S.A. (2015) 112:E5503–12. doi: 10.1073/pnas.1508736112 PMC4603482 26392541

[34] Zheng YWL Chan KCA Sun H Jiang P Su X Chen EZ. Nonhematopoietically derived DNA is shorter than hematopoietically derived DNA in plasma: A transplantation model. Clin Chem. (2012) 58:549–58. doi: 10.1373/clinchem.2011.169318 22052939

[35] Bloom RD Bromberg JS Poggio ED Bunnapradist S Langone AJ Sood P. Cell-Free DNA and active rejection in kidney allografts. J Am Soc Nephrol. (2017) 28:2221–32. doi: 10.1681/ASN.2016091034 PMC5491290 28280140

[36] Liu F Lei L Haitao L Liping S Longkai P. The level of plasm donor-derived cell-free DNA in kidney transplant patients with severe pneumonia. In: Transplantation. (2020) 104:S317. doi: 10.1111/ctr.13652

[37] Whitlam JB Ling L Skene A Kanellis J Ierino FL Slater HR. Diagnostic application of kidney allograft-derived absolute cell-free DNA levels during transplant dysfunction. Am J Transplant. (2019) 19:1037–49. doi: 10.1111/ajt.15142 30312536

[38] Xiao H Gao F Pang Q Xia Q Zeng X Peng J. Diagnostic Accuracy of Donor-derived Cell-free DNA in Renal-allograft Rejection: A Meta-analysis. Transplantation. (2021) 105:1303–10. doi: 10.1097/tp.0000000000003443 32890130

[39] Bunnapradist S Homkrailas P Ahmed E Fehringer G Billings PR Tabriziani H. Using both the fraction and quantity of donor-derived cell-free DNA to detect kidney allograft rejection. J Am Soc Nephrol. (2021) 32:2439–41. doi: 10.1681/ASN.2021050645 PMC8722815 34162734

[40] Gohh R Jordan S Woodward RN Clark-Langone KM Morlan J Silva B. Absolute Quantification of Donor-Derived Cell-Free DNA Does Not Provide Additional Discriminating Power Over Donor-derived Cell-Free DNA Fraction for Detection of Allograft Rejection. Am J Transplant. (2022) 22. https://atcmeetingabstracts.com/abstract/absolute-quantification-of-donor-derived-cell-free-dna-does-not-provide-additional-discriminating-power-over-donor-derived-cell-free-dna-fraction-for-detection-of-allograft-rejection/. Accessed August 28, 2024.

[41] Shen J Guo L Lei W Liu S Yan P Liu H. Urinary donor-derived cell-free DNA as a non-invasive biomarker for BK polyomavirus-associated nephropathy. J Zhejiang Univ Sci B. (2021) 22:917–28. doi: 10.1631/jzus.B2100131 PMC8593525 34783222

[42] Saukkonen K Lakkisto P Pettilä V Varpula M Karlsson S Ruokonen E. Cell-free plasma DNA as a predictor of outcome in severe sepsis and septic shock. Clin Chem. (2008) 54:1000–7. doi: 10.1373/clinchem.2007.101030 18420731

[43] Huang T Yang Z Chen S Chen J. Predictive value of plasma cell-free DNA for prognosis of sepsis. Zhonghua Wei Zhong Bing Ji Jiu Yi Xue. (2018) 30:925–8. doi: 10.3760/cma.j.issn.2095-4352.2018.010.003 30439309

[44] Bunnapradist S Gauthier PM Tabriziani H Swenerton R Ahmed E McKanna T. Case Series: Systemic Infection Alters Background Cell-Free DNA and Influences Results of Donor-Derived Cell-Free DNA Transplant Rejection Assays: PO2398. J Am Soc Nephrol. (2020) 31:728. doi: 10.1681/ASN.20203110S1728a

[45] Reusing JO Yoo J Desai A Brossart K McCormick S Malashevich AK. Association between total cell free DNA and SARS-coV-2 in kidney transplant patients: A preliminary study. Transplant Proc. (2022) 54:1446–54. doi: 10.1016/j.transproceed.2022.02.027 PMC8920956 35618524

[46] García Moreira V Prieto García B de la Cera Martínez T Álvarez Menéndez FV. Elevated transrenal DNA (cell-free urine DNA) in patients with urinary tract infection compared to healthy controls. Clin Biochem. (2009) 42:729–31. doi: 10.1016/j.clinbiochem.2008.12.021 19166828

[47] Burnham P Dadhania D Heyang M Chen F Westblade LF Suthanthiran M. Urinary cell-free DNA is a versatile analyte for monitoring infections of the urinary tract. Nat Commun. (2018) 9:2412. doi: 10.1038/s41467-018-04745-0 29925834 PMC6010457

[48] Morita E Nakashima A Asao H Sato H Sugamura K. Human parvovirus B19 nonstructural protein (NS1) induces cell cycle arrest at G 1 phase. J Virol. (2003) 77:2915–21. doi: 10.1128/jvi.77.5.2915-2921.2003 PMC149759 12584315

[49] Bonvicini F Bua G Conti I Manaresi E Gallinella G. Hydroxyurea inhibits parvovirus B19 replication in erythroid progenitor cells. Biochem Pharmacol. (2017) 136:32–9. doi: 10.1016/j.bcp.2017.03.022 28377277

[50] Verhoeven JGHP Boer K Peeters AMA Clahsen-van Groningen MC Roodnat JI van de Wetering J. A novel high-throughput droplet digital PCR-based indel quantification method for the detection of circulating donor-derived cell-free DNA after kidney transplantation. Transplantation. (2022) 106:1777–86. doi: 10.1097/TP.0000000000004078 35283452

[51] Hinojosa RJ Chaffin K Gillespie M Villarreal VH. Donor-derived cell-free DNA may confirm real-time response to treatment of acute rejection in renal transplant recipients. Transplantation. (2019) 103:E61. doi: 10.1097/TP.0000000000002579 30747838

[52] Walczak K Stawski R Perdas E Brzezinska O Kosielski P Galczynski S. Circulating cell free DNA response to exhaustive exercise in average trained men with type I diabetes mellitus. Sci Rep. (2021) 11:4639. doi: 10.1038/s41598-021-84201-0 33633280 PMC7907132

[53] Haller N Helmig S Taenny P Petry J Schmidt S Simon P. Circulating, cell-free DNA as a marker for exercise load in intermittent sports. PloS One. (2018) 13:e0191915. doi: 10.1371/journal.pone.0191915 29370268 PMC5784997

[54] Al-Hatamleh MAI Tengku MA Alshajrawi OM Ilyas MN Rao SK Majid L. Obesity leads to elevated level of circulating cell-free DNA. Curr Trends BioMed Eng Biosci. (2018) 16:1–4. doi: 10.19080/ctbeb.2018.16.555944

[55] Yan L Chen Y Zhou J Zhao H Zhang H Wang G. Diagnostic value of circulating cell-free DNA levels for hepatocellular carcinoma. Int J Infect Dis. (2018) 67:92–7. doi: 10.1016/j.ijid.2017.12.002 29229500

[56] Uva PD Papadimitriou JC Drachenberg CB Toniolo MF Quevedo A Dotta AC. Graft dysfunction in simultaneous pancreas kidney transplantation (SPK): Results of concurrent kidney and pancreas allograft biopsies. Am J Transplant. (2019) 19:466–74. doi: 10.1111/ajt.15012 29985562

[57] Stratta RJ Farney A Rogers J Orlando G Reeves-Daniel A Mena-Gutierrez A. Utilization of donor-derived cell-free DNA testing in kidney transplantation: do one month values have any prognostic significance? Am J Transplant. (2021) 21. https://atcmeetingabstracts.com/abstract/utilization-of-donor-derived-cell-free-dna-testing-in-kidney-transplantation-do-one-month-values-have-any-prognostic-significance/. Accessed August 28, 2024.

[58] Yoo A Qian I Riedel A Cazac C Bartosic A Bromberg JS. Baseline levels of dd-cf dna after pancreas transplantation: using dd-cfdna as an indicator for pancreas rejection and biopsy avoidance. Am J Transplant. (2021) 21. https://atcmeetingabstracts.com/abstract/baseline-levels-of-dd-cf-dna-after-pancreas-transplantation-using-dd-cfdna-as-an-indicator-for-pancreas-rejection-and-biopsy-avoidance/. Accessed August 28, 2024.

[59] Ventura-Aguiar P Ramirez-Bajo MJ Rovira J Bañón-Maneus E Hierro N Lazo M. Donor-derived cell-free DNA shows high sensitivity for the diagnosis of pancreas graft rejection in simultaneous pancreas-kidney transplantation. Transplantation. (2022) 106:1690–7. doi: 10.1097/TP.0000000000004088 PMC9311279 35289777

[60] Zhao D Zhou T Luo Y Wu C Xu D Zhong C. Preliminary clinical experience applying donor-derived cell-free DNA to discern rejection in pediatric liver transplant recipients. Sci Rep. (2021) 11:1138. doi: 10.1038/s41598-020-80845-6 33441886 PMC7807012

[61] Levitsky J Kandpal M Guo K Kleiboeker S Sinha R Abecassis M. Donor-derived cell-free DNA levels predict graft injury in liver transplant recipients. Am J Transplant. (2022) 22:532–40. doi: 10.1111/ajt.16835 34510731

[62] Anand S Lopez-Verdugo F Sanchez-Garcia J Dong L Fife M Krong J. Longitudinal variance of Donor-Derived Cell-Free DNA (dd-cfDNA) in Stable Kidney Transplant (KTx) patients are influenced by donor/recipient variables. Clin Transplant. (2021) 35:e14395. doi: 10.1111/ctr.14395 34165192

[63] Agbor-Enoh S Chan JL Singh A Tunc I Gorham S Zhu J. Circulating cell-free DNA as a biomarker of tissue injury: Assessment in a cardiac xenotransplantation model. J Heart Lung Transplant. (2018) 37:967–75. doi: 10.1016/j.healun.2018.04.009 PMC6707066 29933912

[64] Dhayat SA Yang Z. Impact of circulating tumor DNA in hepatocellular and pancreatic carcinomas. J Cancer Res Clin Oncol. (2020) 146:1625–45. doi: 10.1007/s00432-020-03219-5 PMC7256092 32338295

[65] Chopra B Sureshkumar KK. Emerging role of cell-free DNA in kidney transplantation. World J Exp Med. (2021) 11:55–65. doi: 10.5493/WJEM.V11.I5.55 34877265 PMC8611196

[66] Paul RS Almokayad I Collins A Raj D Jagadeesan M. Donor-derived cell-free DNA: advancing a novel assay to new heights in renal transplantation. Transplant Direct. (2021) 7:E664. doi: 10.1097/TXD.0000000000001098 33564715 PMC7862009

[67] Melancon JK Khalil A Lerman MJ. Donor-derived cell free DNA: is it all the same? Kidney360. (2020) 1:1118–23. doi: 10.34067/KID.0003512020 PMC8815488 35368782

[68] Harkins KM Schaefer NK Troll CJ Rao V Kapp J Naughton C. A novel NGS library preparation method to characterize native termini of fragmented DNA. Nucleic Acids Res. (2021) 48:e47. doi: 10.1093/NAR/GKAA128 PMC7192605 32112100

[69] Zhou Q Liu F Guo L Chen R Yuan X Li C. A novel urine cell-free DNA preservation solution and its application in kidney transplantation. Nephrology. (2021) 26:684–91. doi: 10.1111/nep.13884 33866646

